# Gut Microbiota of Wild and Captive Alpine Musk Deer (*Moschus chrysogaster*)

**DOI:** 10.3389/fmicb.2019.03156

**Published:** 2020-01-21

**Authors:** Yewen Sun, Yujiao Sun, Zhihui Shi, Zhensheng Liu, Chang Zhao, Taofeng Lu, Hui Gao, Feng Zhu, Rui Chen, Jun Zhang, Ruliang Pan, Baoguo Li, Liwei Teng, Songtao Guo

**Affiliations:** ^1^Shaanxi Key Laboratory for Animal Conservation, Northwest University, Xi’an, China; ^2^College of Wildlife and Protected Area, Northeast Forestry University, Harbin, China; ^3^College of Food and Biological Engineering, Henan University of Animal Husbandry and Economy, Zhengzhou, China; ^4^Key Laboratory of Wildlife Conservation, China State Forestry Administration, Harbin, China; ^5^Institute of Laboratory Animal Science, Guizhou University of Traditional Chinese Medicine, Guiyang, China; ^6^School of Human Sciences, The University of Western Australia, Perth, WA, Australia; ^7^Harbin Veterinary Research Institute, Chinese Academy of Agricultural Sciences, Harbin, China

**Keywords:** wild musk deer, gut microbiota, short-chain fatty acid, Firmicutes to Bacteroides/Prevotella (F/B) ratio, methane

## Abstract

As for the wild animals, their diet components are always changed, so that we have to monitor such changes by analyzing the modification of intestinal microbial community. Such effort allows us to amend their conservation strategies and tactics accordingly so that they are able to appropriately adapt to the new environment and dietary selection. In this study we focus on the gut flora of two groups of an endangered species, Alpine musk deer (*Moschus chrysogaster*), wild group (WG) which is compared with that of the individuals of the same species but kept in the captivities (CG), a control group. Such a project is aimed to work out whether the composition of the gut microbes has significantly been changed due to captive feedings. To do so, we used 16S rRNA amplicon sequencing to characterize gut bacteria of the musk deer from the two groups. The results show that there is a significant difference in community structure of the bacteria: WG shows significant enrichment of Firmicutes and depletion of Bacteroidetes, while CG has a significant abundance of Proteobacteria and Euryarchaeota. Metagenomics was used to analyze the differences in functional enzymes between the two groups. The related results indicate that genes in WG are mostly related to the enzymes digesting cellulose and generating short-chain fatty acids (SCFAs) for signaling pathways, but CG shows enrichment in methanogenesis, including the CO_2_/H_2_ pathway and the methylotrophic pathway. Thus, this study indicates that the Firmicutes-rich gut microbiota in the WG enables individuals to maximize their energy intake from the cellulose, and has significant abundance of Euryarchaeota and methanogenesis pathways that allow them to reduce redundant energy consumption in methane metabolism, ensuring them to adapt to the wild environments.

## Introduction

Intestinal microbiology of the mammals is frequently shaped by many factors, including dietary selection, phylogenic development, and environmental modifications (Turnbaugh et al., 2009; Yatsunenko et al., 2012; Schnorr et al., 2014; Clemente et al., 2015). Among them, the changes in dietary selection have been considered to be able to rapidly affect the composition of gut microbiota (Wu et al., 2011; David et al., 2014; Wang et al., 2019). Thus, artificial feeding wild animals during the program of *ex situ* conservation have to be modified on their dietary selection adopted in the wild, despite humans attempt to simulate wild diets for them. In other words, there exist a great variety in the richness of the dietary components between the animal groups living in the wild and the captivities. Thus, it is critical to monitor digestive system of the animals in the captivities in order to know whether they have adapted to artificial food provisioned and new environment – an important issue in wildlife conservation (Murray et al., 2016). Within the digestive system, intestinal microbiome plays an important role in digestion and absorption of the food, and maintaining animals’ health (Ley et al., 2005; Flint et al., 2008; Gupta et al., 2011; Smith et al., 2013). The distal intestine harbors billions of bacteria for those functions. Members of the Bacteroidetes and Firmicutes divisions dominate the microbiota in mammalian gut and participate in colonic metabolism for undigested food remains, mostly fibers, by a complex metabolic energy-harvesting mechanism based on cross-feeding and co-metabolism (Eckburg et al., 2005; Candela et al., 2010). Some studies have shown that the ratio of Firmicutes to Bacteroidetes (F/B) indicates the amount of the energy absorbed; such a ratio in the body with the obesity is higher than normal individuals (Ley et al., 2006; Samuel and Gordon, 2006; Fabrice et al., 2009; Fernandes et al., 2014). A study on the associations between gut microbes and nutrient absorption in humans has presented stool energy loss in lean individuals, and a 20% increase of Firmicutes and a corresponding decrease of Bacteroidetes are associated with an increased energy harvest of 150 kcal (Jumpertz et al., 2011).

Compared with the carnivores, gastric and intestinal tracts of the ruminants are rich in symbiotic bacteria that helps the body digest plant fibers (Kohl et al., 2011; Muegge et al., 2011; Saro et al., 2012; Kim et al., 2013). The development of a rumen is driven by close interactions among the rumens, metabolic products of microorganisms, diet and the host (Li et al., 2012). Gut microbiota catabolizes the fibers not completely hydrolyzed by host enzymes, in addition to utilizing polysaccharides as an energy source (Flint et al., 2008). Glycans are processed by the distal gut microbiota, generating biologically significant short-chain fatty acids (SCFAs, predominantly acetate, butyrate, and propionate), which serve as the principal energy source for colonocytes (Topping and Clifton, 2001). Fibers may be involved in the regulation of food intake and energy balance via the SCFA-mediated modulation of the secretion of gut hormones (Freeland and Wolever, 2010). An inverse association was observed through the study of between Bacteroidetes counts and body mass index values; there is a significant positive association between F/B ratios and SCFA concentrations, and such an association between Bacteroidetes counts and SCFA concentrations is, however, significantly negative (Fernandes et al., 2014).

Rumens show significant functional variation in gut symbiotic microbiota due to a series of different habitats. Ruminants in high-altitude, such as Yaks (*Bos grunniens*) and sheep (*Ovis aries*), show significantly lower levels of methane and higher yields of volatile fatty acids (VFAs) than their low-altitude relatives, for instance cattle (*Bos taurus*) and sheep (*O. aries*) (Zhang et al., 2016). Methane is a byproduct of methanogens in the fermentation process, and high methane production leads to more energy loss. A high level of methane production leads to more energy loss. Rumens can lose about 2–12% of the energy caused by methane products referring to alternative diets (Johnson and Ward, 1996). At the same time, methane production is negatively correlated with VFA production in ruminants – the increase in VFA production could greatly inhibit the production of methane due to the competing for hydrogen through methane-producing pathway (Zhang et al., 2016; Moraïs and Mizrahi, 2019). In the studies on herbivorous primates, such as western lowland gorillas (*Gorilla gorilla*), chimpanzees (*Pan troglodytes*), and black howler monkeys (*Alouatta pigra*), it is indicated that gut symbiotic microbiota also changes seasonally in responding to seasonal dietary variation, which helps the hosts improve energy intake efficiency during the food shortage period (Amato et al., 2015; Gomez et al., 2015; Hicks et al., 2018). Such phenomenon allows the hosts to adapt to environmental changes under the principles and regulations of natural selection and environmental adaptation.

It is reported that some wild animal populations raised by the artificial program to avoid declining population size have to change their digestive system following the modification of dietary components that are different from those taken from the wild. Different from nature food, the proportion of carbohydrate is relatively high in provisioned food. This kind of artificial food feeding is based on previous experience rather than on the diet of wild populations (Xie, 2011). The CG diet, different from those from the wild, has a higher proportion of carbohydrate, but lower level of fibers (Supplementary Tables 1, 2).

Control group usually faces a high risk of disease, especially gastrointestinal ones which have a high mortality rates (Yan et al., 2016). Mostly gastrointestinal problems are associated with intestinal flora disorder in forest musk deer (Zhou et al., 2016). A research on musk deer indicates that there has been an increased proportion of Bacteroides and Prevotella in captive individuals (Li et al., 2017). Another research on captive primates reports that, due to dietary changes, especially the decreased fiber components, primates lost substantial portions of signature microbiota in captivity, and they were colonized by human-associated gut bacterial genera of Bacteroides and Prevotella, which lead to a decreased gut microbiota diversity (Clayton et al., 2016), implying an increased risk of intestinal disorders (Moeller et al., 2014).

Alpine musk deer (*Moschus chrysogaster*) are rapidly disappearing in the wild. It is the endangered species on the list of Red List by IUCN, as such China has kept some of them in captivity. We are, however, facing the problems of how to maintain appropriate food components that can keep their normal nutrition adopted in the wild, and a healthy digestive system naturally selected through environmental adaptation (Zhu et al., 2012; Xu et al., 2014; Murray et al., 2016; Yan et al., 2016). China’s WGs in winter face a server challenge due to the scarce food covered by snow, although it is not confirmed that they face famine in nutrition and energy demand. Compared to the wild condition, the captive group obviously is provisioned more food and even high energy items, but diversity of provisioned food seems to be less than the nature food. Diet of captive group contains less fiber than that of WG. That is to say that CG groups avoid the problem of famine but potentially face more issues relevant to poor or inappropriate nutrition, and digestive disorder (Carding et al., 2015) under the circumstance with artificially provisioned food, which has occurred to many species of the caged animals (Flinchum, 1997; Knapp et al., 2013). Thus, the standardized levels of nutritional requirement and fiber components in the feeding program are critically required for the deer in the captivities. In this regard, the microbiota of wild group can provide a good indicator that tells the direction of provision artificial food (Gorvitovskaia et al., 2016; Hale et al., 2017). Thus, in this study we are going to provide some guidelines through a study focusing on the comparison of gut microbiota between the WG and the CG, in order to understand how symbiotic gut microbes in CG have been changed in energy absorption and transformation due to provisioned food and how gut microbiota has responded to such a transaction of food chains. The results will be tangibly used to make or amend the conservation strategies and tactics for the musk deer.

## Experimental Procedures

### Fecal Sample Collection

Thirty-seven fecal samples were collected totally: fourteen from 14 individuals in Xinglongshan Alpine musk deer farm and 23 from 23 wild individuals in the Mts. Helan National Nature Reserve, China. The records on dietary components and antibiotic usage of the formers were from the Xinglongshan Alpine musk deer farm, the biggest captive group in China where two feeding bases are allocated (Supplementary Figure 1). The estimated population size of wild group is less than 100 individuals (Yang et al., 2003). Fecal samples were collected by researchers immediately after defecation and the samples were immediately stored in a centrifuge tube and preserved at −80°C until extraction of genomic DNA. Fecal collection tools are sterile cotton swabs and sterile toothpicks. Only fresh fecal samples are collected from the fecal center to avoid environmental contamination on the surface.

### DNA Extraction and Sequencing of 16S rRNA Gene Amplicons

DNA samples were extracted from approximately 300 mg of each fecal samples (sample size *n* = 37) with a modified protocol of the DNA Stool Mini Kit (QIAGEN, Hilden, Germany). The PCR amplifications were carried out with Phusion^®^ High-Fidelity PCR Master Mix. Specific primers for identification of bacteria were 515F (5′-GTGBCAGCMGCCGCGGTAA-3′) and 806R (5′-GGACTACHVGGGTWTCTAAT-3′). Sequencing libraries were generated using a TruSeq^®^ DNA PCR-Free Sample Preparation Kit (Illumina, United States) following the manufacturer’s instructions, and index codes were added. The library quality was assessed using a Qubit 2.0 Fluorometer (Thermo Scientific) and an Agilent Bioanalyzer 2100 system. Finally, the library was sequenced on an Illumina HiSeq 2500 platform, and 250 bp paired-end reads were generated.

### Determining OTU and Taxonomy Assignments

Paired-end reads were merged with FLASH (V1.2.7) software^1^, after which the raw tags were analyzed under specific filtering conditions to obtain high-quality clean tags according to the QIIME quality control processes (V1.7.0)^2^. They were compared with the reference database^3^ and the chimera sequences were removed to get the Effective Tags finally.

Sequence analysis was performed with Uparse software (Uparse v7.0.1001)^4^. Sequences with ≥97% similarity were assigned to the same OTU. A representative sequence for each OTU was screened for further annotation.

### Statistical Analysis

Considering the small sample size of both groups (CG *n* = 14, WG *n* = 23), the data collected may not be normally distributed. Thus, a non-parametric statistics, the Wilcox test, was used to analyse the differences between the two groups (Supplementary Table 3). A significant level is set at *p* < 0.05. Pearson’s correlations were used to test the associations between variables with a normal distribution; for non-normally distributed data, Spearman’s rank test was used. The differences with a *p*-value < 0.05 (two-tailed) is considered to be statistically significant. A principal component analysis (PCA) and a principal coordinate analysis (PCoA) were implemented in the R “ggplot2” package to evaluate whether the gut microbiota structure is significantly segregated across the cohorts. The QIIME software (Version 1.7.0) was used to calculate the UniFrac distance and construct the unweighted pair-group method with arithmetic mean (UPGMA) clustering trees. To identify the biomarkers showing statistically significant differences between the two groups, an LDA Effect Size (LEfSe) analysis was performed with LEfSe software and a default setting for the LDA Score of 4.

### Metagenomic Sequencing

We randomly select one fecal sample from CG and another three from WG three individuals (samples size, *n* = 4). A total of 1 μg of DNA per sample was used as input material for the DNA sample preparations. Sequencing libraries were generated using the NEBNext^®^ Ultra DNA^TM^ Library Prep Kit for Illumina (NEB, United States) following the manufacturer’s recommendations. After cluster generation, the library preparations were sequenced on an Illumina HiSeq 2500 platform, and paired-end reads were generated.

### Gene Functional Classification

To classify functions of the sequences, unigenes were compared to the databases using the DIAMOND software; the highest match result (one HSP > 60 bits) was selected. From the results of comparison, the relative abundances of different functional levels were counted based on three databases: Kyoto Encyclopedia of Genes and Genomes (KEGG) (Kanehisa et al., 2004), Evolutionary Genealogy of Genes: Non-supervised Orthologous Groups (eggNOG) (von Mering et al., 2005), and Carbohydrate-Active enzymes Database (CAZy) (Cantarel et al., 2009).

## Results

### 16S rRNA Gene Surveying Reveal Hierarchical Separation of Two Musk Deer Groups

To characterize bacterial lineages presented in the fecal microbiota of the musk deer, we performed multiplex pyrosequencing of the 16S rRNA gene with the Illumina HiSeq 2500 platform, and generated a dataset consisting of 1 589 579 filtered high-quality classifiable 16S rRNA gene sequences with a mean average (± SD) of 42 962 ± 16 458 sequences per sample (Supplementary Table 4). Using a minimum identity of 97% as the threshold for any sequence pair, we identified 37 999 bacterial operational taxonomic units (OTUs) with an average of 1 027 OTUs per sample, 14 of which were previously undescribed (Supplementary Figure 2 and Supplementary Table 4).

**FIGURE 1:**
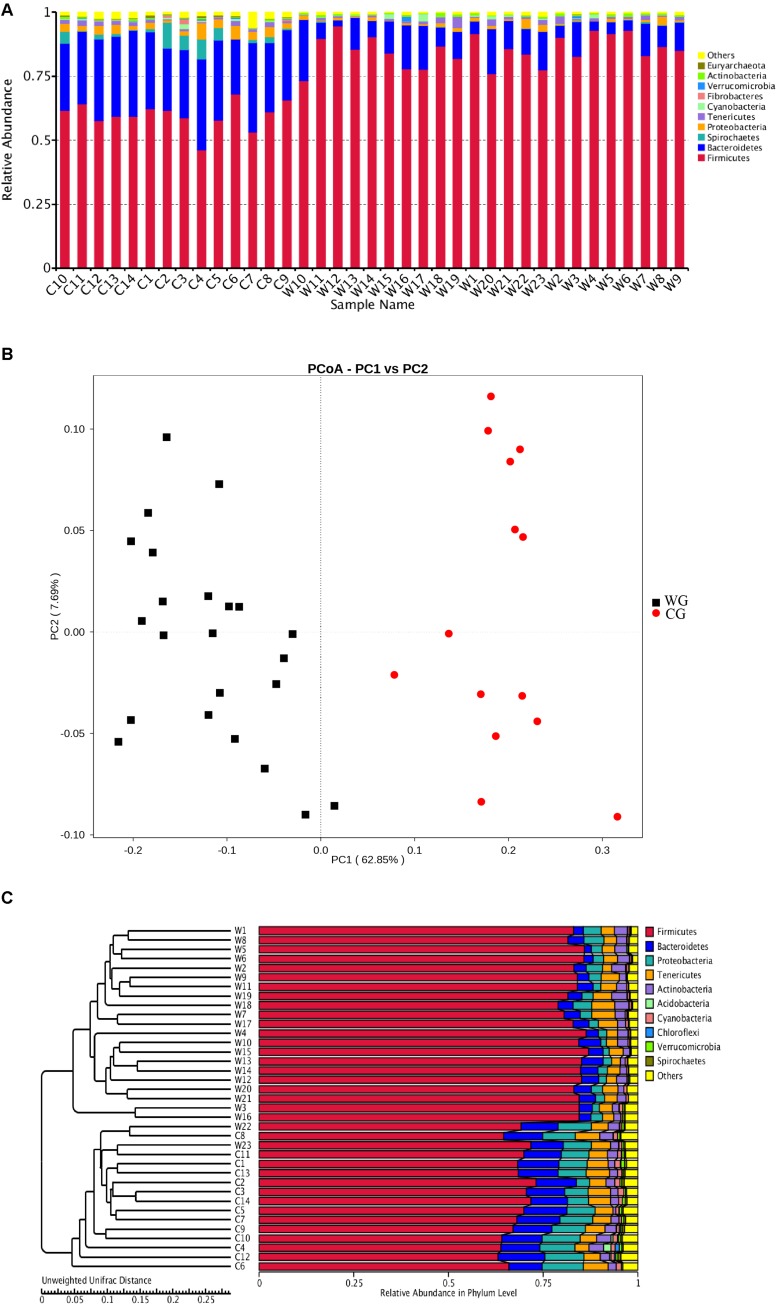
Both the structure of flora and the unweighted UniFrac distance analysis reveal hierarchical separation of two musk deer groups. **(A)** Bacterial OTUs from 37 musk deer gut communities classified to the phylum level. Bars for each library represent the percentage of species assigned to each phylum with 80% bootstrap confidence. **(B)** Weighted UniFrac PCoA, which shows structural segregation between the WG and CG cohorts (C for CG, W for WG). Symbols representing individual communities are colored (black: WG, red: CG). **(C)** UPGMA tree based on the Unweighted UniFrac distance method. The tree on the left side shows the distance of each sample; the graph on the right side shows the relative abundance of the species at the phylum level.

We found that musk deer shared similar main community constituents, both wild and domesticated groups. Firmicutes formed the most numerous microbial communities in the gut, followed by Bacteroidetes. However, the two groups can be clearly separated based on the structure of the gut microbiota. For the 10 most abundant phyla, in WG and CG microbiota, respectively, were presented here: Firmicutes, 85.27 and 59.80%; Bacteroidetes, 10.30 and 29.23%; Proteobacteria, 1.17 and 3.14%; Spirochetes, 0.11 and 2.98%; Tenericutes, 1.26 and 1.20%; Cyanobacteria, 0.49 and 0.48%; Fibrobacteres, 0.03 and 0.30%; Verrucomicrobia, 0.14 and 0.11%; Actinobacteria, 0.61 and 0.42%; and Euryarchaeota, 0.06 and 0.32% (Figure 1A). Unsupervised clustering with PCoA and PCA indicates the variation in our dataset in in which WG microbiota is clustered from CG microbiota along principal coordinate 1 (Figure 1B and Supplementary Figure 3). Such significant structural segregation of the gut microbiota across the two groups is also confirmed by unweighted pair-group method with arithmetic mean (UPGMA) tree analysis, a method that measures similarity among microbial communities based on the degree to which their component taxa coincide (Figure 1C).

### Taxonomic Differences of Musk Deer Gut Microbiota

**FIGURE 2:**
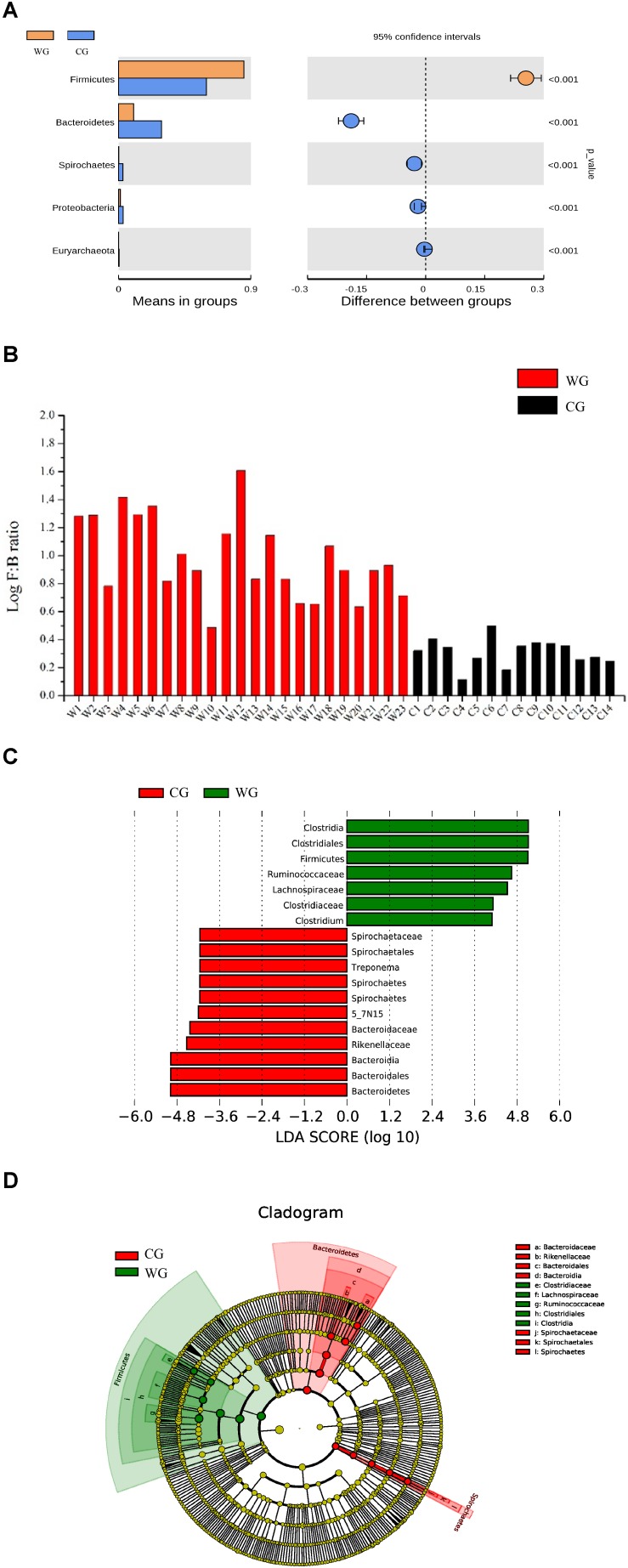
Bacterial taxa showing significant differences between the two groups as detected by Wilcoxon test and LEfSe analysis. **(A)** Species with a significant difference (*p* < 0.05) at the phylum level based on the Wilcoxon test between the two groups. **(B)** Log F/B ratios in CG and WG individuals are clearly different (red: WG, black: CG). **(C,D)** Distribution histogram of LDA and system evolutionary distribution based on biomarkers with statistically significant differences in abundance between the two groups.

Statistical analysis with Wilcoxon test indicates that Firmicutes (*p* < 0.001) and Bacteroidetes (*p* < 0.001) are significantly differentiated between the WG and the CG. This result is strengthened by using the Benjamini and Hochberg false discovery rate to correct the *p* values, which again shows significant discriminating factors in Firmicutes (3.51 × 10^–13^), Bacteroidetes (5.68 × 10^–13^), Spirochetes (7.8 × 10^–3^), Proteobacteria (6.8 × 10^–4^), and Euryarchaeota (7.1 × 10^–4^) (Figure 2A and Supplementary Table 3a). Intestinal flora of the two groups share a similar structure, with the most populous components of anaerobic bacteria from the Bacteroidetes and Firmicutes phyla. However, there is a prominent difference in the ratio of Firmicutes to Bacteroidetes between the two groups: the WG group has fewer Bacteroidetes and more Firmicutes than the CG group (Figure 2B). In the WG group, the ratio of Firmicutes to Bacteroidetes varies from 3.06 to 40.42, while that for CG group is from 1.31 to 3.17 (Supplementary Table 5).

We also used Wilcoxon test to identify the genera showing significant differences at the 95% confidence level (Supplementary Table 3b). Among the 19 genera we identified 9 showing higher mean proportions in the WG group. The genera with high abundance in the WG group are concentrated in Firmicutes. Except for the genus *Bifidobacterium* that belongs to Actinobacteria, all the genera belong to Firmicutes, including *Clostridium*, *Roseburia*, *Dorea*, *Blautia*, and *Faecalibacterium*. However, for the C group, the genera at higher levels mostly belong to Bacteroidetes, including *5-7N15*, *Bacteroides*, *Paludibacter*, *Prevotella*, and *Paraprevotella* (Supplementary Figure 4).

To identify the characteristics of differential abundance and the associated categories of the intestinal flora, we used LEfSe (LDA Effect Size) (Score > 4) to seek biomarkers showing significant differences between the groups, referring to statistical tests and on biological relevance (Figures 2C,D). The results were consistent with the Wilcoxon test; the biomarkers with higher richness in the WG group, including *Clostridiaceae, Lachnospiraceae, Ruminococcaceae, Clostridiales, Clostridia*, and *Faecalibacterium*, all belonging to Firmicutes, whereas the biomarkers with higher richness in the CG group belonging to Bacteroidetes and Spirochetes.

### Differences in Metabolic Pathways of Microbes Between the Two Groups

To explore the differences in microbial functions between the two groups, we analyzed gene functional classification in a metagenomic analysis of fecal samples from four musk deer to focus on the functions of the communities. We then compared the catalog with the KEGG and the CAZy databases to assess the differences in functional capacities presented in bacteria. KEGG metabolic pathways provide a highly integrated picture of global gut cell metabolism, suggesting that the gut microbiome of musk deer has enriched activity for metabolism of carbohydrates (gene number: 26 522), amino acids (21 370), nucleotides (15 586), and energy (12 759) (Supplementary Figure 5).

Based on the KEGG database, we counted the unique enzyme reactions of each group. The reactions of intestinal flora from WG are represented by K06045 (squalene-hopene/tetraprenyl-beta-curcumene cyclase), K01047 (secretory phospholipase A2), K00036 (glucose-6-phosphate 1-dehydrogenase), K00832 (aromatic-amino-acid transaminase), K00022 (3-hydroxyacyl-CoA dehydrogenase) and K01692 (enoyl-CoA hydratase) (Supplementary Figure 4). These enzymes are mainly involved in biological processes, such as metabolism of terpenoids and polyketone compounds; biosynthesis of sesquiterpenoids and triterpenoids; synthesis and metabolism of SCFAs and biosynthesis of phenylalanine, tyrosine, and tryptophan. Stilbenoids, which are often found in the trees of the pine family, belonging to the family of phenylpropanoids and sharing most of their biosynthetic pathway with chalcones (Sobolev et al., 2006). Triterpenoids possess a rich chemistry and pharmacology (e.g., cholesterol) with several pentacyclic motifs, and some of these compounds show promise as anti-cancer agents (Andre et al., 2016). All of the compounds mentioned above are important components of the metabolic pathways of the WG. Meanwhile, for the CG group, most of the specialized enzymatic reactions were related to glycolysis/gluconeogenesis and hydroxymethylglutaryl-CoA synthase (Supplementary Figure 6).

**FIGURE 3:**
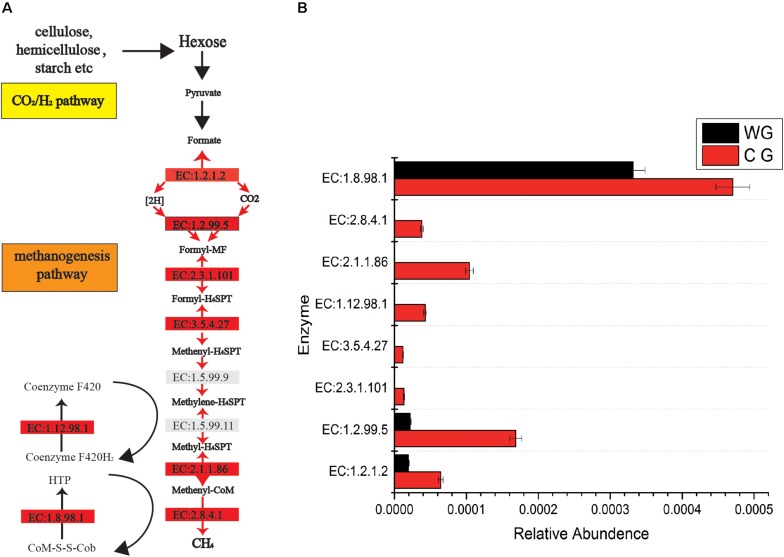
Comparisons of gene and transcript abundance for enzymes involved in WG and CG. **(A)** Diagram of Co_2_/H_2_ methanogenesis pathway shows enzymes involved in each biochemical reaction. **(B)** Relative abundance for each enzyme between WG and CG.

In addition, CG are significantly rich in methanogenesis pathways (Figure 3). A total of 46 enzymes were identified in the CG and WG, 27 of which are shared between each other, 17 enzymes are exclusively in the CG, and only 2 enzymes are unique to the WG. Among the enzymes involved in each biochemical reaction in the CO_2_/H_2_ pathway, 1.2.1.2, 1.2.99.5 are common to both groups, but the abundance in CG is higher. The other five major enzymes 2.8.4.1, 2.3.1.101, 3.5.4.27, 2.1.1.86, 1.12.98.1 are unique to CG. Enzyme 2.8.4.1 is the final and rate-limiting step in the catalytic CH4 biogenesis (Cedervall et al., 2010).

**FIGURE 4:**
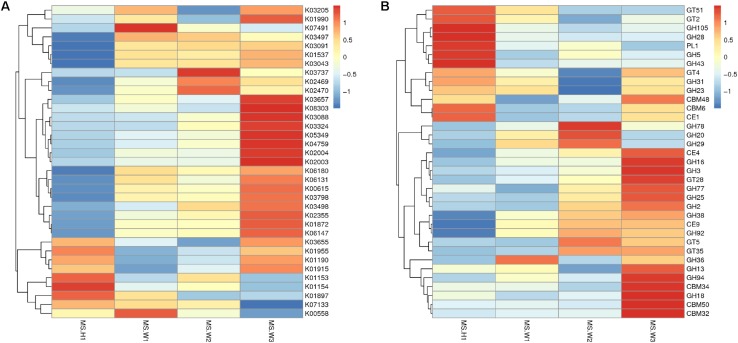
Differences in the functional profiles of fecal microbiomes in the two musk deer populations (normalized by *Z*-score across all datasets). KEGG and CEs that showed the largest differences, in proportional representation, between CG and WG populations. **(A)** Heat map of the top 35 functions based on the KEGG database. **(B)** Heat map of the top 35 functions based on the CAZy database.

To detect the functional differences in intestinal flora among all the individuals, we built a clustering heat map based on the 35 functions with the highest abundance (Figure 4A). These results show that the WG gut microbiome has more enriched activity for metabolism of energy than does the CG gut microbiome; the signal pathways involves include ABCB-BAC (K06147) and the Ca^2+^-transporting ATPases (K01537, EC 3.6.3.8).

In total, 4 883 genes were annotated to 37 glycosyl transferase families (GTs), and the major enzymes involved are cellulase synthase, chitinase, phosphodiester D-mannose aminotransferase, glucosyltransferase, hyaluronan synthase, and chitosan oligosaccharide synthase. Among all carbohydrate esterases (CEs), the gene showing the highest abundance in the WG microbiota is CE4, accounting for 32.9% of the total number of annotated genes in this family. Abundance cluster analysis of carbohydrate-active enzymes shows that CEs involved in the metabolism of chito-oligosaccharide are more abundant in the WG group (CE4, CE9), whereas glycoside hydrolases (GHs) involved in the metabolism of starch and disaccharides (EC 3.2.1.20, EC 3.2.1.22, EC 3.2.1.24) are mainly represented in CG microbes (Figure 4B).

## Discussion

### Effects of Taxonomic Differences on Musk Deer Energy Intake and Health

Bacteroidetes and Firmicutes are the most abundant phyla between the WG group and the CG group of the Alpine musk deer. The former has more Firmicutes, while the latter displays more Bacteroidetes. The genera with high abundance in the WG group are concentrated in Firmicutes and most of them have the ability to degrade fiber, including *Clostridium*, *Roseburia*, *Dorea*, *Blautia*, and *Faecalibacterium*. While, regarding the captive group, the genera at higher levels are mostly belonged to Bacteroidetes, including *5-7N15*, *Bacteroides*, *Paludibacter*, *Prevotella*, and *Paraprevotella*.

The community constituent of the CG is similar to that reported from other ruminants, and Firmicutes form the most abundant microbial community in the gut, followed by Bacteroidetes (Ley et al., 2008; Brulc et al., 2009), and a similar result has previously been reported for another species in the same genus, the forest musk deer (*Moschus berezovskii*) (Li et al., 2017). The WG group has more Firmicutes, while the CG group expresses more Bacteroidetes, especially Bacteroides and Prevotella. Such result is also similar to that reported on captive primates, due to dietary changes, especially the decreased fiber components, microbiota in captive primates become colonized by human-associated gut bacterial genera Bacteroides and Prevotella (Clayton et al., 2016).

Dietary habits are considered as one of the main factors contributing to the diversity of gut microbiota, and taxonomic shifts in microbiome composition corresponding to dietary variation (Backhed et al., 2005). The two musk deer populations share a similar climate, so that we classify the factors under farm breeding, such as diet and sanitation as human intervention. The WG in the Helan Mountains are well protected from illegal hunting by nature reserve, so the largest obstacle to their survival in winter is food shortage. The diets of WG reveal a diverse diet spectrum, mostly consisting of leaves and branches containing high fibers, whereas CG’s diet has a regular recipe mainly containing carrot, corn, dry leaves and fodder, they have higher level of fat and simple carbohydrates, low in fibers compared to that of WG (Supplementary Tables 1, 2). The cellulose contents taken by the WG are approximately three times higher than that in the CG. Different dietary intake and microbial profiles may result in altered colonic fermentation patterns, leading to different fecal SCFA concentrations. Such differences in composition and proportion also lead to the changes in metagenomic function and energy intake of the Alpine musk deer.

Therefore, the ability to absorb nutrients from high-fiber foods is the key to the survival of wild musk deer, and high F/B ratios in gut microbiota are clearly of great significance. WG shows higher F/B ratios in this regard. A study on the associations between gut microbes and nutrient absorption in humans has shown stool energy loss in lean individuals, such as a 20% increase in Firmicutes and a corresponding decreased of Bacteroidetes are associated with an increased energy harvest of 150 kcals (Jumpertz et al., 2011). It has been suggested that a higher F/B ratio may be associated with the increased energy harvest from colonic fermentation and the increased production of SCFAs (Ley et al., 2006). Referring to the F/B ratio, it is possible that the energy intake capacity of WG is better than CG.

The genera found in WG, such as *Clostridium*, *Ruminococcaceae*, *Roseburia*, *Oscillospira*, *Anaerostipes*, *Dorea*, *Blautia*, and *Faecalibacterium*, can degrade fibers to produce organic acids and SCFAs (Koh et al., 2016). For example, *Phascolarctobacterium* species produce propionate via succinate fermentation (Dot et al., 1993), *Blautia* is known an acetogen (Park et al., 2012), *Faecalibacterium* and *Roseburia* are butyrate producers (Duncan et al., 2002; Holmstrøm et al., 2004). SCFAs, mainly including acetate, propionate and butyrate, are the major anions in the gut, and are rapidly absorbed by colonic epithelial cells. A large amount of acetate enters systemic circulation, mainly from peripheral tissues for lipid production; propionate is mainly consumed by the liver for gluconeogenesis; butyrate is the preferred source of energy for colon cells (Pomare et al., 1985; Scott et al., 2008). Having comprehensively analyzed the structure of nutrients and bacteria we speculate that the higher abundance of flora are associated with digestive fiber increasing rate of nutrient uptake from the food in WG, possibly by generating more SCFAs, which are thought to be responsible for approximately 50–70% of the energy supply for ruminants (Bergman, 1990). The significant enrichment of fiber-degrading bacteria in the wild population corresponds to its higher fiber feeding, indicating that the intestinal microbes play an active role in helping the host to absorb nutrients and adapt to the environment.

**FIGURE 5:**
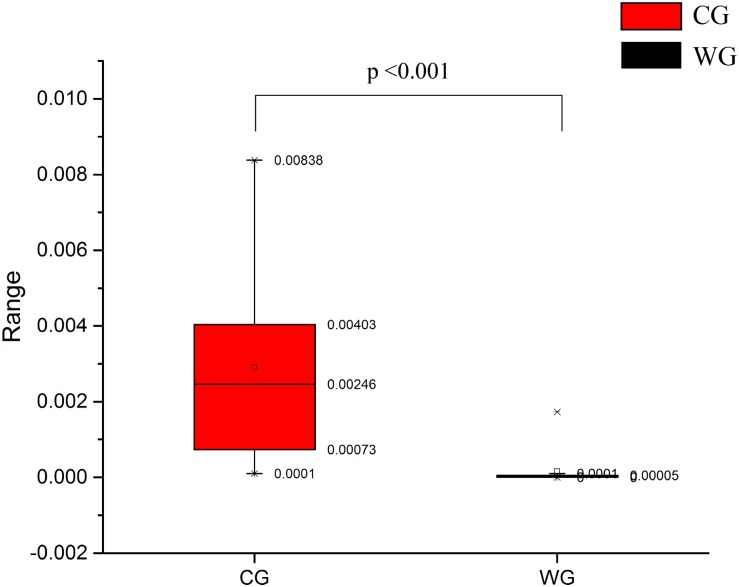
Comparison of methanobacteriaceae relative abundance between CG and WG.

High abundance genera in the CG, *Prevotella* and *Bacteroides* have been considered to be associated with diet-related gut microbial enterotypes in humans (Wu et al., 2011). *Prevotella* is a common bacterial genus capable of degrading hemicellulose, pectin and simpler carbohydrates, such as those expected in fruits and low-complexity fibrous resources (Russell and Baldwin, 1979). CG has high abundance in Euryarchaea and methanogens. The abundance of methanobacteriaceae in CG is significantly higher than WG (Figures 2A, 5), and Methanobacteria is methanogens (Liu and William, 2008). As a byproduct of rumen fermentation of methanogens, methane results in energy loss (Morgavi et al., 2010).

The species richness and structure of the intestinal microbiota can often reflect the health status of the body, and disease may be caused by abnormal flora, while some species help resist the occurrence of some intestinal diseases. Butyrate generated by fiber degradation is related to the decreasing of inflammation in the body (Wolever et al., 1989; Scheppach, 1994; De Filippo et al., 2010; Zhang et al., 2015). Butyrate and propionate can protect against colorectal cancer and inflammation, at least partly by inhibiting histone deacetylases HDACs (Johnstone, 2002; Flint et al., 2012). Another important biomarker of CG, *Treponema*, is a genus of spiral-shaped bacteria, whose subspecies are responsible for diseases such as syphilis, bejel, and yaws (Antal et al., 2002). The investigation of captive forest musk deer shows that gastrointestinal diseases are the most common and have high mortality rates, reaching approximately 30% (Li et al., 2017). The high incidence of gastrointestinal diseases in CG may be related to intestinal flora. Proteobacteria were also significantly enriched in CG which is considered as a marker of intestinal disorders (Shin et al., 2015).

### Metagenomic Function and Energy Intake of Musk Deer

Metabolic pathways with higher abundance in WG include ABCB-BAC (K06147) and the Ca^2+^-transporting ATPases (K01537, EC 3.6.3.8). The Ca^2+^-transporting ATPases are P-type ATPases that undergo covalent phosphorylation during the transport cycle. This enzyme family comprises three types of Ca^2+^-transporting enzymes that are found in the plasma membrane, the sarcoplasmic reticulum and in yeast. Ca^2+^ is transported from the cytosol into the sarcoplasmic reticulum in muscle cells (Inesi et al., 1982; MacLennan et al., 1997). ABCB-BAC is an ATP-binding cassette, subfamily B. The ATP-binding cassette (ABC) transporter superfamily is one of the largest gene families and encodes a functionally diverse group of membrane proteins involved in the energy-dependent transport of a wide variety of substrates across membranes (Dean and Allikmets, 1995). Subfamily B is considered the only human subfamily to have both half and full types of transporters. ABCB1 was discovered as a protein overexpressed in certain drug-resistant tumor cells, and cells that overexpress this protein exhibit multi-drug resistance (Dean et al., 2001). Exporters or effluxers, which are present in both prokaryotes and eukaryotes, function as pumps that extrude toxins and drugs out of the cell (Davidson et al., 2008).

Focusing on the metabolic pathways in the CG group, we found that most of the highly expressed or unique compounds shown in the KEGG pathways are associated with glucose metabolism. Hydroxymethylglutaryl-CoA synthase is an enzyme that catalyzes the reaction in which acetyl-CoA condenses with acetoacetyl-CoA to form 3-hydroxy-3-methylglutaryl-CoA (HMG-CoA) (Theisen et al., 2004), which is an intermediate in both cholesterol synthesis and ketogenesis (Ruiz-Albert et al., 2002). This reaction is over-activated in patients with diabetes mellitus type I if left untreated. The CG group shows significant overrepresentation of glycosyltransferase pathways, mainly related to their high-carbohydrate diet.

In addition, the enrichment of methane metabolic pathways in CG is directly related to increased methane production (Shi et al., 2014). Macromolecular hydrolysis and microbial fermentation produce large amounts of hydrogen, which, if accumulated in an uncontrolled manner, inhibits microbial metabolism and prevents metabolic cascades (Flint, 1997; Hobson and Stewart, 2012). The use of hydrogen is mainly carried out by methanogens (archaea) and acetogens, as well as microorganisms that reduce sulfate and nitrate to a lesser extent (Morvan et al., 1996; Müller, 2003; Lin et al., 2011). Enrichment of methane metabolic pathways and the increase of methanogens in captive individuals, show that captive individuals are more dependent on methane metabolic pathways, while wild individuals are enriched in acetogens and functional gene deletions in methane production pathways, showing more dependence on acetogens for hydrogen utilization. SCFAs are valuable for the energy requirements of the host, while methane and its retained energy are emitted into the atmosphere. From the wild to the captivity, the changes in the flora function caused by different diet directly lead to an increase in the level of methane metabolism, which affects the efficiency of the energy absorption.

Before we studied the intestinal flora of musk deer, we had investigated the feeding habits of the two musk deer groups. Unlike the fixed diet of CG, the WG has many more choices. In addition to high-nutrition food, they are also willing to eat plants containing secondary metabolites and minerals, which are beneficial for health. In fact, many of the plants which they prefer are herbs, but most of those plants have not been identified. Although we only studied the feeding habits and intestinal flora of the WG during one season, many unique enzymatic reactions shown on the KEGG signaling pathways are associated with secondary metabolites in plants and provide some clues. We found many traces suggesting excavation of fungi by musk deer (Supplementary Figure 7). As a primary component of cell walls in fungi (Tang et al., 2015), chitin is thought to be able to activate the innate immune system through eosinophils or macrophages, as well as an adaptive immune response through T helper cells (Elieh Ali Komi et al., 2017). Acetyl xylan esterase and chitin deacetylase have the function to digest the cell walls of fungi and plants, chitin in fungal cell walls, and dermatoplasm in bacterial cell walls. The high abundance of chitinase in the WG intestinal flora is related to their fungal diet; it also confirmed the correlation between the function of intestinal microbe flora and diet.

Referring to the results found in this study, it is clear that the impacts caused by different dietary selection and living environment, which have resulted in different intestinal flora of musk deer, are remarkable. Although intestinal flora has a certain degree of the plasticity in helping the host adapt to the changes from natural to artificial dietary supplies, some potential health risks to captive populations cannot be ignored; such as the declining nutrient absorption efficiency and the increasing of potential pathogenic bacteria. During breeding period the individuals in the captivities require a variety of dietary components. Meanwhile the proportion of dietary fibers should be appropriately increased to help musk deer maintain a stable and health intestinal flora under artificial environment. To improve captivity-based breeding programs more experiments are still required to search for ideal ways of narrowing the gap between wild and captivity group in terms of dietary selection, which would play in important role in musk deer conservation.

## Data Availability Statement

The datasets generated for this study can be found in the NCBI/BioProject/PRJNA380002 (https://www.ncbi.nlm.nih.gov/bioproject/PRJNA380002).

## Ethics Statement

The animal study was reviewed and approved by the Ethics Committee of Northwest University.

## Author Contributions

SG and LT conceived and designed the experiments. YeS, YuS, ZS, and ZL carried out the DNA extraction and data analysis. CZ, TL, HG, FZ, and RC participated in the sample collection. SG, YeS, YuS, and JZ wrote the manuscript. RP and BL assisted with experiments and advice on manuscript content. All authors read and approved the final manuscript.

## Conflict of Interest

The authors declare that the research was conducted in the absence of any commercial or financial relationships that could be construed as a potential conflict of interest.

## Footnotes

1 http://ccb.jhu.edu/software/FLASH/

2 http://qiime.org/index.html

3 http://drive5.com/uchime/uchime_download.html

4 http://drive5.com/uparse/
