# Supplementary figures and images for: Gut Microbiota of Wild and Captive Alpine Musk Deer (Moschus chrysogaster)
Source: Front Microbiol. 2020 Jan 21;10:3156. doi: 10.3389/fmicb.2019.03156 (PMC6985557; doi:10.3389/fmicb.2019.03156)

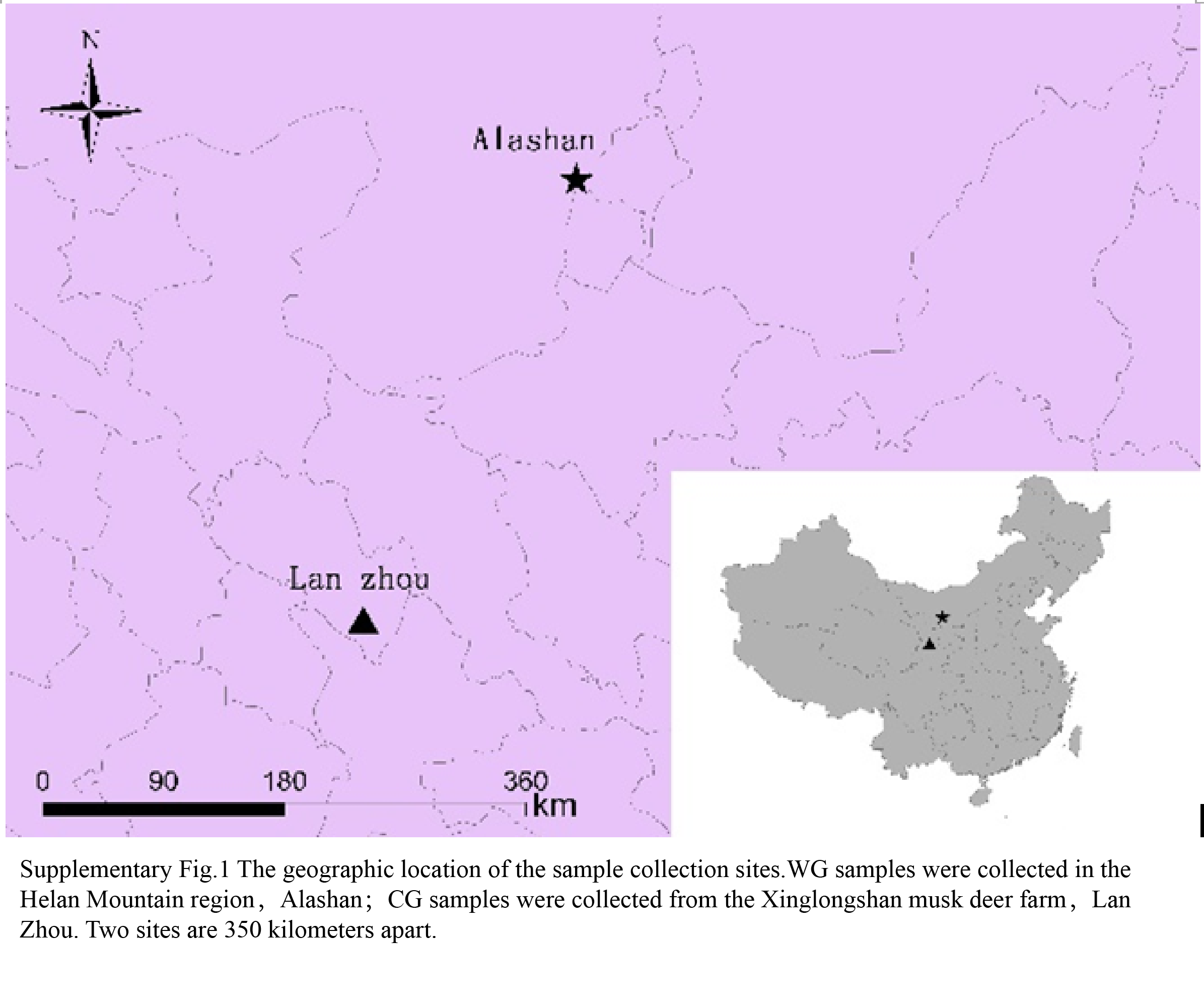

Supplement: Supplementary file 1 [file Image_1.PNG]

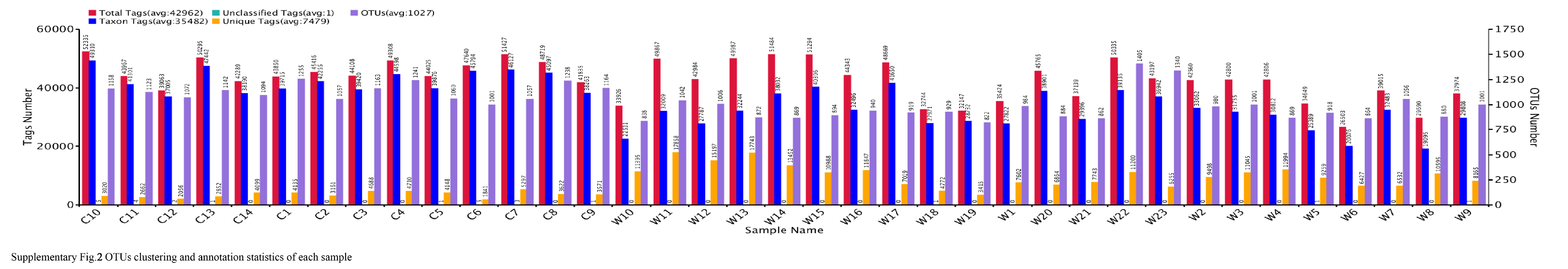

Supplement: Supplementary file 2 [file Image_2.png]

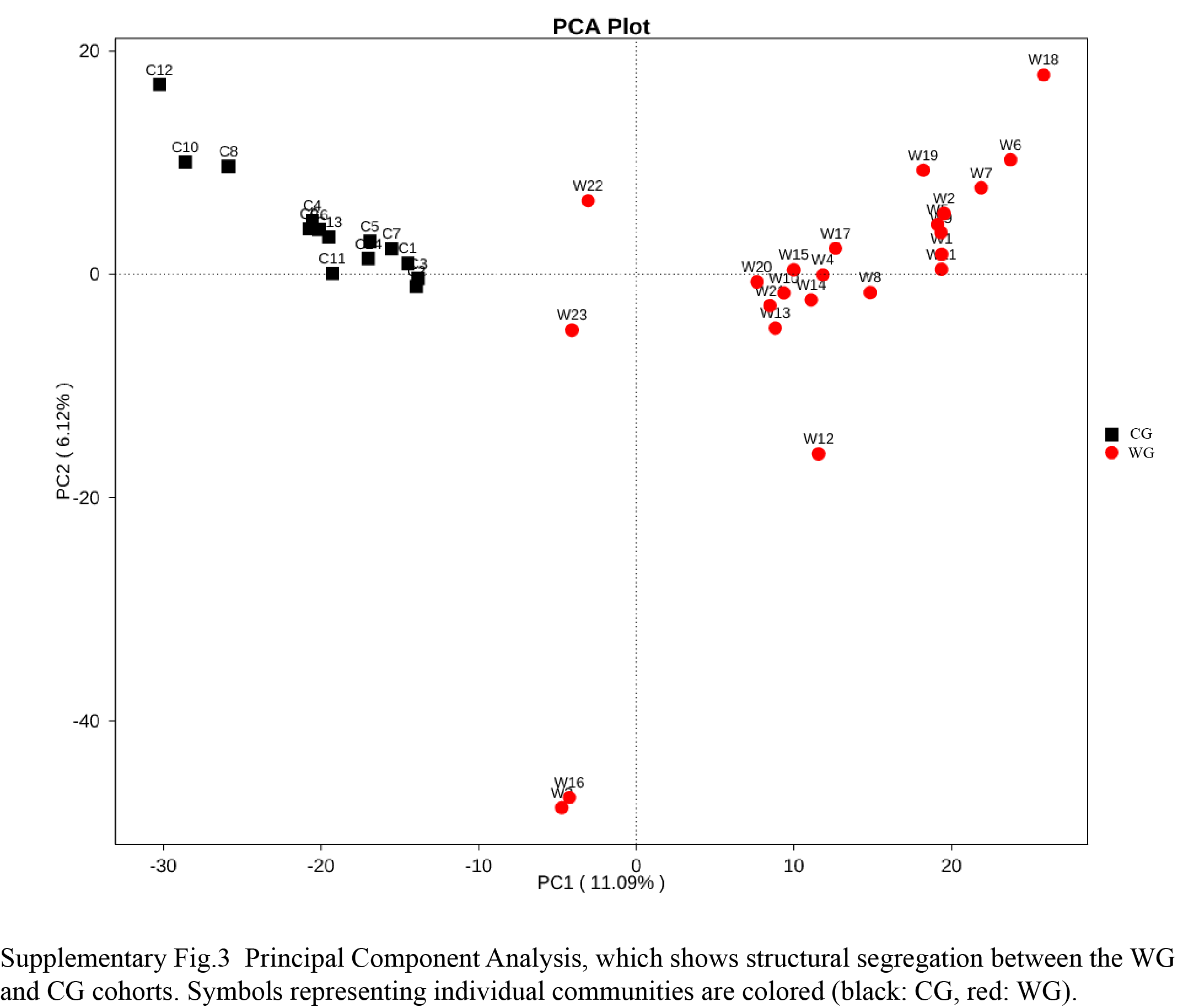

Supplement: Supplementary file 3 [file Image_3.PNG]

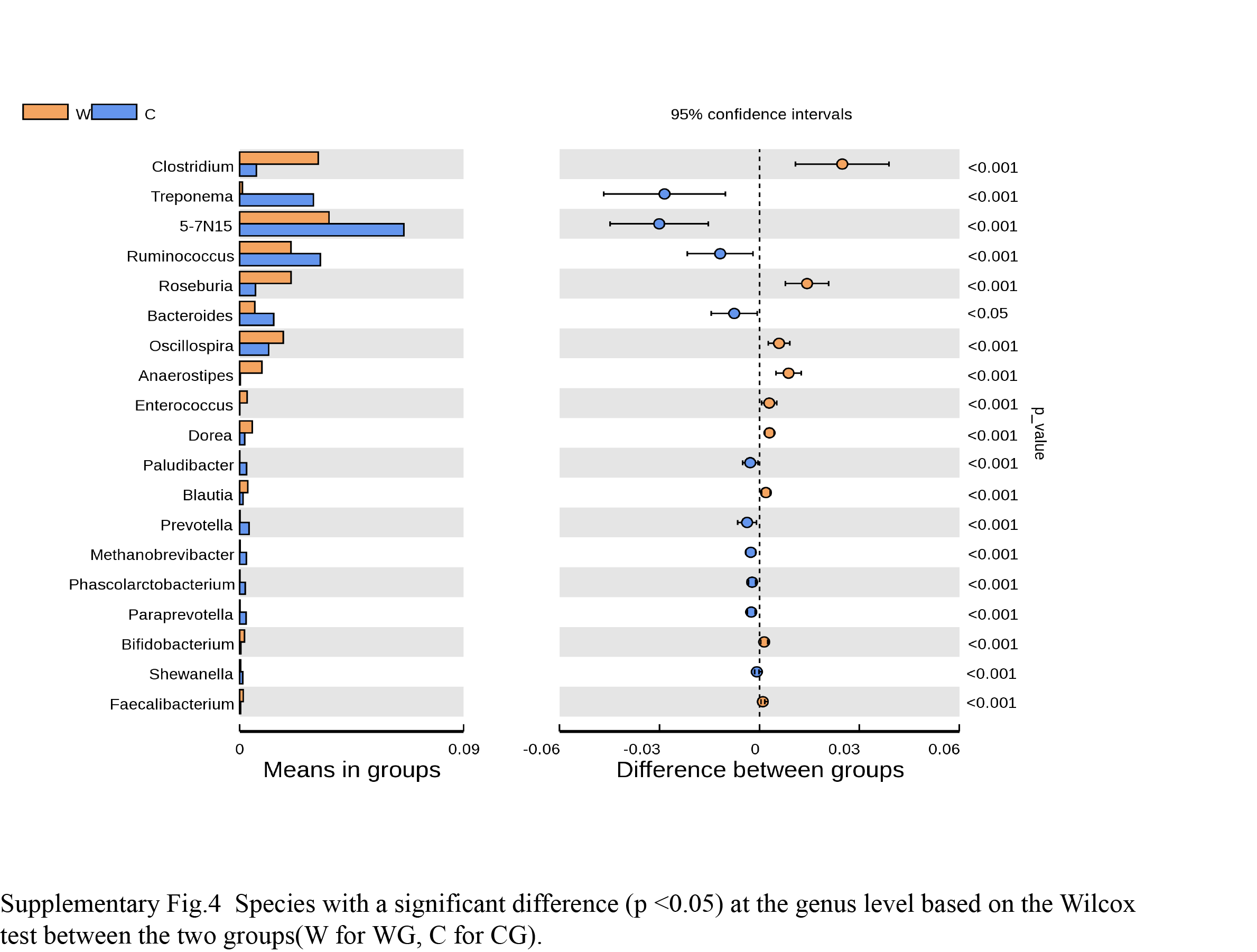

Supplement: Supplementary file 4 [file Image_4.PNG]

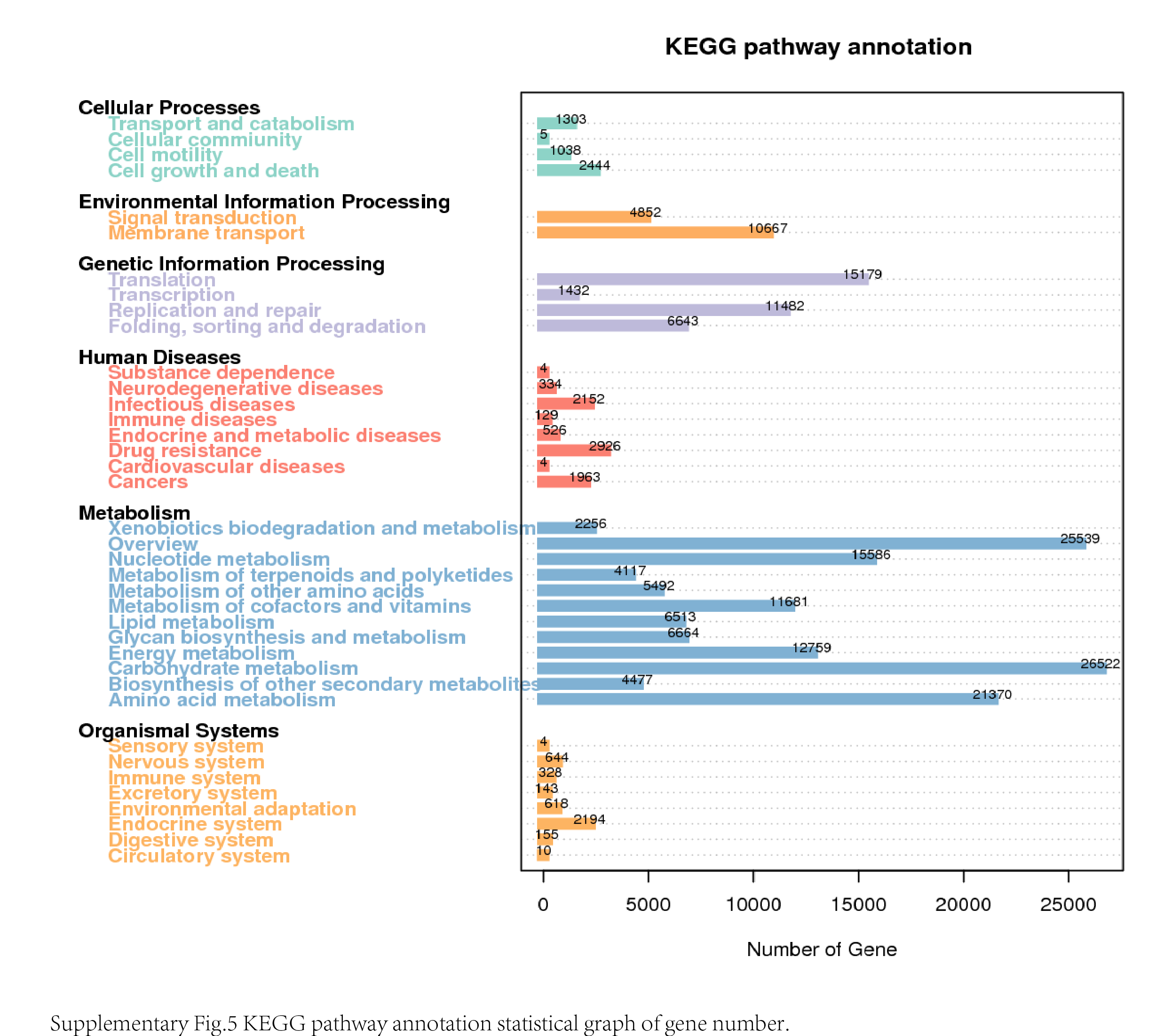

Supplement: Supplementary file 5 [file Image_5.PNG]

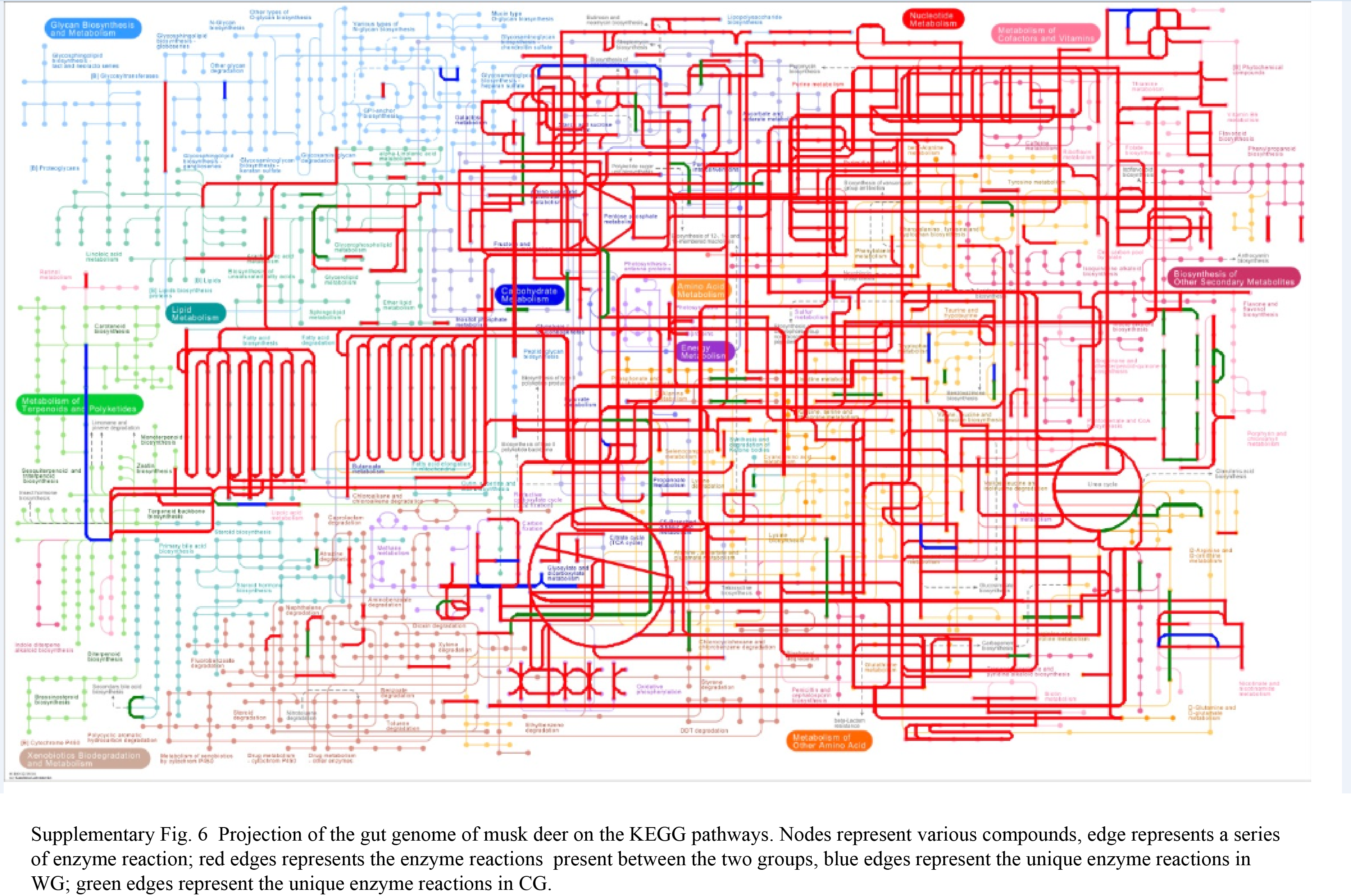

Supplement: Supplementary file 6 [file Image_6.PNG]

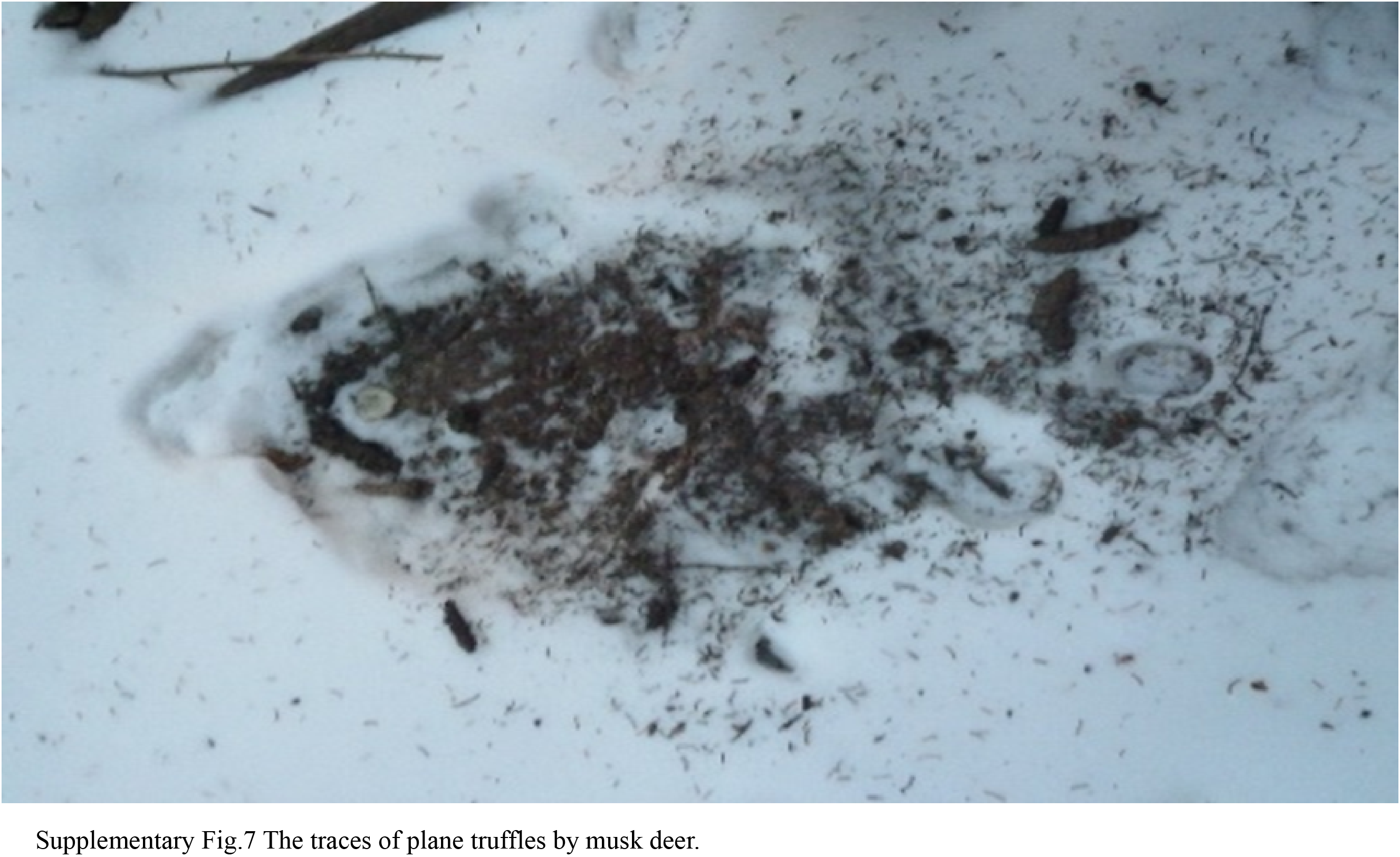

Supplement: Supplementary file 7 [file Image_7.PNG]
