# Supplementary material for: Gut Microbiota of Wild and Captive Alpine Musk Deer (Moschus chrysogaster)
Source: Front Microbiol. 2020 Jan 21;10:3156. doi: 10.3389/fmicb.2019.03156 (PMC6985557; doi:10.3389/fmicb.2019.03156)
Supplement: Supplementary file 8 [file Data_Sheet_1.docx]

**Supplementary material**

Table. 1 The ratio and nutritional content of plants eaten by wild musk deer (*Moschus sifanicus*) of Helan Mountain in winter.

| **Plant species** | **Frequeny** | **Crude protein(%)** | **Ether extract(g/kg)** | **Fiber (%)** |
| --- | --- | --- | --- | --- |
| *Picea crassifolia* | 4.77 | 3.78 | 7.63 | 22.07 |
| *Juniperus rigida* | 4.63 | 4.93 | 31.38 | 45.55 |
| *Sabina vulgaris* | 0.06 | 6.21 | 40.15 | 32.04 |
| *Salix characta* | 1.09 | 3.15 | 9.17 | 22.18 |
| *Ostryopsis davidiana* | 2.39 | 10.90 | 29.34 | 13.77 |
| *Urtica dioica* | 3.54 | 10.66 | 12.84 | 12.40 |
| *Berberis caroli* | 1.25 | 9.35 | 24.54 | 38.35 |
| *Ribes pulchellum* | 1.3 | 4.81 | 24.45 | 29.49 |
| *Spiraea tomentulosa* | 0.31 | 4.75 | 27.09 | 37.27 |
| *Cotoneaster acutifolius* | 1.13 | 4.61 | 2.34 | 38.84 |
| *Pentaphylloides parvifolia* | 0.37 | 22.91 | 2.13 | 62.59 |
| *Pentaphylloides davurica* | 2.53 | 5.27 | 44.72 | 34.91 |
| *Sanguisorba alpina* | 3.97 | 9.86 | 19.27 | 27.04 |
| *Prunus sibirica* | 4.92 | 3.89 | 15.62 | 27.75 |
| *Prunus mongolica* | 3.93 | 5.73 | 9.76 | 53.54 |
| *Caragana stenophylla* | 4.31 | 10.81 | 2.60 | 36.74 |
| *Astragalus chingianus* | 11.33 | 6.12 | 11.44 | 46.14 |
| *Syringa oblata* | 2.06 | 4.14 | 3.60 | 50.13 |
| *Nepeta sibirica* | 2.13 | 8.36 | 2.11 | 38.27 |
| *Leptodermis ordosica* | 6.47 | 3.45 | 13.54 | 47.22 |
| *Lonicera edulis* | 13.68 | 5.48 | 13.17 | 15.49 |
| *Lonicera microphylla* | 2.96 | 4.91 | 14.19 | 29.10 |
| *Erigeron acer* | 0.01 | 8.63 | 6.27 | 18.24 |
| *Saussurea alaschanica* | 2.04 | 7.82 | 10.54 | 37.86 |
| *Dendranthema chanetii* | 1.48 | 0.89 | 49.26 | 35.52 |
| *Roegneria barbicalla* | 5.08 | 8.31 | 4.32 | 45.92 |
| *Carex pediformis* | 10.93 | 6.33 | 6.22 | 44.36 |
| *Tuber mdicum* | 0.69 | 25.55 | 85.71 | 12.11 |
| *Geopora cooperi* | 0.01 | 31.61 | 18.03 | 18.30 |
| *Cortinarius purpurascens* | 0.62 | 20.77 | 7.23 | 17.42 |

Table. 2 .Nutritional content of the main food of captive musk deer per 100 grams.

| **Species** | **Protein(%)** | **Crude fiber(%)** | **Carbohydrate(%)** | **Fat(%)** |
| --- | --- | --- | --- | --- |
| **Carrot** | 0.8 | 2 | 5 | 0 |
| **Corn** | 8 | 6.4 | 78 | 3.8 |
| **Soya bean** | 35 | 15.5 | 25 | 13 |
| **wheat bran** | 15 | 31 | 61 | 4 |
| **Apricot leaves** | 13.2 | 9.8 | 52.2 | 5.7 |
| **Elm leaves** | 6.6 | 11.3 | 48.7 | 5.0 |
| **Acacia leaves** | 19.6 | 15.2 | 42.7 | 2.4 |
| **Peach leaves** | 13.7 | 12.4 | 52.6 | 6.2 |

***** **The nutrient contents of all the substances listed above are for reference only, as there is a big difference of the** **nutrient contents for one species between different regions and different varieties of plants. All of our data are from researches.**
