# Supplementary material for: Gut Microbiota of Wild and Captive Alpine Musk Deer (Moschus chrysogaster)
Source: Front Microbiol. 2020 Jan 21;10:3156. doi: 10.3389/fmicb.2019.03156 (PMC6985557; doi:10.3389/fmicb.2019.03156)
Supplement: Supplementary file 9 [file Data_Sheet_2.docx]

**Supplementary material**

| **Taxonomy** | **W** | ***p*-value** |
| --- | --- | --- |
| Firmicutes | 0 | < 0.01 |
| Bacteroidetes | 321 | < 0.01 |
| Spirochaetes | 292 | < 0.01 |
| Proteobacteria | 288 | < 0.01 |
| Euryarchaeota | 315.5 | < 0.01 |

Table. 3a. The results of Wilcox test between the CG and WG groups in Phylum level top5.

Table 3b. The results of Wilcox test between the CG and WG groups in Genus level.

| **Taxonomy** | **W** | ***p*-value** |
| --- | --- | --- |
| Clostridium | 37 | < 0.01 |
| Treponema | 292.5 | < 0.01 |
| 57-N15 | 265 | < 0.01 |
| Ruminococcus | 248 | < 0.01 |
| Roseburia | 15 | < 0.01 |
| Bacteroides | 234 | <0.05 |
| Oscillospira | 68 | <0.01 |
| Anaerostipes | 1 | <0.01 |
| Enterococcus | 65 | <0.01 |
| Dorea | 32.5 | <0.01 |
| Paludibacter | 303 | <0.01 |
| Blautia | 50 | <0.01 |
| Prevotella | 295 | <0.01 |
| Methanobrevibacter | 310.5 | <0.01 |
| Phascolarctobacterium | 317 | <0.01 |
| Paraprevotella | 312 | <0.01 |
| Bifidobacterium | 44.5 | <0.01 |
| Shewanella | 278 | <0.01 |
| Faecalibacterium | 53.5 | <0.01 |

Table 3c.The results of Wilcox test between the CG and WG groups in F/B ratio.

| **F/B ratio** | **W** | ***p*-value** |
| --- | --- | --- |
| CG vs WG | 1 | < 0.01 |
