# Supplementary material for: Gut Microbiota of Wild and Captive Alpine Musk Deer (Moschus chrysogaster)
Source: Front Microbiol. 2020 Jan 21;10:3156. doi: 10.3389/fmicb.2019.03156 (PMC6985557; doi:10.3389/fmicb.2019.03156)
Supplement: Supplementary file 10 [file Data_Sheet_3.docx]

| Sample  Name | Total  tag | Taxon  Tag | Unclassified  Tag | Unique  Tag | OUT_num |
| --- | --- | --- | --- | --- | --- |
| C10 | 52335 | 49310 | 5 | 3020 | 1158 |
| C11 | 43967 | 41301 | 4 | 2662 | 1123 |
| C12 | 39063 | 37005 | 2 | 2056 | 1072 |
| C13 | 50295 | 47442 | 1 | 2852 | 1142 |
| C14 | 42289 | 38190 | 0 | 4099 | 1094 |
| C1 | 43850 | 39715 | 0 | 4135 | 1255 |
| C2 | 45416 | 42255 | 0 | 3161 | 1057 |
| C3 | 44108 | 39420 | 0 | 4688 | 1163 |
| C4 | 49308 | 44598 | 0 | 4710 | 1241 |
| C5 | 44025 | 39876 | 1 | 4148 | 1060 |
| C6 | 47640 | 45794 | 5 | 1841 | 1001 |
| C7 | 51427 | 46127 | 3 | 5297 | 1057 |
| C8 | 48719 | 45097 | 0 | 3622 | 1238 |
| C9 | 41835 | 38263 | 1 | 3571 | 1164 |
| W10 | 33926 | 22531 | 0 | 11395 | 838 |
| W11 | 49867 | 32009 | 0 | 17858 | 1042 |
| W12 | 42984 | 27787 | 0 | 15197 | 1006 |
| W13 | 49987 | 32244 | 0 | 17743 | 872 |
| W14 | 51484 | 38032 | 0 | 13452 | 869 |
| W15 | 51294 | 40306 | 0 | 10988 | 894 |
| W16 | 44343 | 32496 | 0 | 11847 | 940 |
| W17 | 48669 | 41650 | 0 | 7019 | 919 |
| W18 | 32744 | 27971 | 1 | 4772 | 929 |
| W19 | 32147 | 28732 | 0 | 3415 | 822 |
| W1 | 35424 | 27822 | 0 | 7602 | 984 |
| W20 | 45765 | 38901 | 0 | 6864 | 884 |
| W21 | 37139 | 29396 | 0 | 7743 | 862 |
| W22 | 50335 | 39135 | 0 | 11200 | 1405 |
| W23 | 43197 | 36942 | 0 | 6255 | 1340 |
| W2 | 42560 | 33062 | 0 | 9498 | 980 |
| W3 | 42800 | 31755 | 0 | 11045 | 1001 |
| W4 | 42806 | 30812 | 0 | 11994 | 869 |
| W5 | 34649 | 25389 | 1 | 9259 | 918 |
| W6 | 26503 | 20076 | 0 | 6427 | 864 |
| W7 | 39015 | 32483 | 0 | 6532 | 1056 |
| W8 | 29690 | 19095 | 0 | 10595 | 880 |
| W9 | 37974 | 29808 | 1 | 8165 | 1001 |

Supplementary Table.4 OUT clustering and annotion statistics of each sample.
