# Supplementary material for: Gut Microbiota of Wild and Captive Alpine Musk Deer (Moschus chrysogaster)
Source: Front Microbiol. 2020 Jan 21;10:3156. doi: 10.3389/fmicb.2019.03156 (PMC6985557; doi:10.3389/fmicb.2019.03156)
Supplement: Supplementary file 11 [file Data_Sheet_4.docx]

Supplementary Table.5 The ratio of Firmicutes to Bacteroidetes between the two groups.

| Taxonomy | Firmicutes | Bacteroidetes | F/B | LOG10 |
| --- | --- | --- | --- | --- |
| C1 | 0.623723488 | 0.298193244 | 2.091675448 | 0.320494299 |
| C10 | 0.616967793 | 0.261953391 | 2.355257897 | 0.372038469 |
| C11 | 0.642524221 | 0.282429955 | 2.274986093 | 0.356978746 |
| C12 | 0.57674784 | 0.317989002 | 1.813735178 | 0.258573876 |
| C13 | 0.592563498 | 0.313851794 | 1.888036042 | 0.276010281 |
| C14 | 0.592720607 | 0.335689971 | 1.765678627 | 0.24691166 |
| C2 | 0.616967793 | 0.24278607 | 2.54119931 | 0.405038729 |
| C3 | 0.587588374 | 0.265357423 | 2.214328005 | 0.345241953 |
| C4 | 0.462738937 | 0.354438335 | 1.305555556 | 0.115795357 |
| C5 | 0.579313957 | 0.311338047 | 1.860723297 | 0.269681795 |
| C6 | 0.680649385 | 0.214977743 | 3.166138855 | 0.500529957 |
| C7 | 0.53197172 | 0.348939513 | 1.524538496 | 0.183138395 |
| C8 | 0.611050013 | 0.270280178 | 2.26080217 | 0.354262561 |
| C9 | 0.657187745 | 0.274050799 | 2.398050831 | 0.379858385 |
| W1 | 0.917151087 | 0.047865933 | 19.16083151 | 1.282414352 |
| W10 | 0.73197172 | 0.23891071 | 3.063787812 | 0.486258684 |
| W11 | 0.897826656 | 0.062843676 | 14.28666667 | 1.154930912 |
| W12 | 0.946216287 | 0.023409269 | 40.42058166 | 1.606602559 |
| W13 | 0.85435978 | 0.125582613 | 6.803169308 | 0.832711279 |
| W14 | 0.90285415 | 0.064519508 | 13.99350649 | 1.145926554 |
| W15 | 0.840429432 | 0.123959152 | 6.779890156 | 0.831222658 |
| W16 | 0.778214192 | 0.170987169 | 4.551301685 | 0.658135624 |
| W17 | 0.776590731 | 0.172191673 | 4.510036496 | 0.654180056 |
| W18 | 0.868185389 | 0.074260278 | 11.69111425 | 1.067855905 |
| W19 | 0.819586279 | 0.104425242 | 7.848545637 | 0.894789188 |
| W2 | 0.901911495 | 0.046347211 | 19.45988701 | 1.289140314 |
| W20 | 0.75925635 | 0.175962294 | 4.314880952 | 0.634968818 |
| W21 | 0.85833988 | 0.109452736 | 7.842105263 | 0.894432667 |
| W22 | 0.83686829 | 0.097931396 | 8.545454545 | 0.931735168 |
| W23 | 0.775281487 | 0.149358471 | 5.190743338 | 0.715229555 |
| W3 | 0.827022781 | 0.136789736 | 6.045941807 | 0.781463963 |
| W4 | 0.929353234 | 0.035663786 | 26.05873715 | 1.415953365 |
| W5 | 0.916208432 | 0.046713799 | 19.6132287 | 1.292549092 |
| W6 | 0.928934276 | 0.041110238 | 22.59617834 | 1.354034994 |
| W7 | 0.829536528 | 0.126368159 | 6.564442603 | 0.817197855 |
| W8 | 0.864886096 | 0.084262896 | 10.26413922 | 1.011322534 |
| W9 | 0.851898403 | 0.108929039 | 7.820673077 | 0.893244132 |
|  |  |  |  |  |
